# SUMOylation of the Kv4.2 Ternary Complex Increases Surface Expression and Current Amplitude by Reducing Internalization in HEK 293 Cells

**DOI:** 10.3389/fnmol.2021.757278

**Published:** 2021-11-02

**Authors:** Meghyn A. Welch, Leslie-Anne R. Jansen, Deborah J. Baro

**Affiliations:** ^1^Department of Biology, Georgia State University, Atlanta, GA, United States; ^2^Neuroscience Institute, Georgia State University, Atlanta, GA, United States

**Keywords:** dipeptidyl peptidase-like protein, potassium channel interacting protein, Kv4, trafficking, ion channel, SUMOylation, small ubiquitin-like modifier (SUMO), A-type potassium current

## Abstract

Kv4 α-subunits exist as ternary complexes (TC) with potassium channel interacting proteins (KChIP) and dipeptidyl peptidase-like proteins (DPLP); multiple ancillary proteins also interact with the α-subunits throughout the channel’s lifetime. Dynamic regulation of Kv4.2 protein interactions adapts the transient potassium current, IA, mediated by Kv4 α-subunits. Small ubiquitin-like modifier (SUMO) is an 11 kD peptide post-translationally added to lysine (K) residues to regulate protein–protein interactions. We previously demonstrated that when expressed in human embryonic kidney (HEK) cells, Kv4.2 can be SUMOylated at two K residues, K437 and K579. SUMOylation at K437 increased surface expression of electrically silent channels while SUMOylation at K579 reduced IA maximal conductance (Gmax) without altering surface expression. KChIP and DPLP subunits are known to modify the pattern of Kv4.2 post-translational decorations and/or their effects. In this study, co-expressing Kv4.2 with KChIP2a and DPP10c altered the effects of enhanced Kv4.2 SUMOylation. First, the effect of enhanced SUMOylation was the same for a TC containing either the wild-type Kv4.2 or the mutant K437R Kv4.2, suggesting that either the experimental manipulation no longer enhanced K437 SUMOylation or K437 SUMOylation no longer influenced Kv4.2 surface expression. Second, instead of decreasing IA Gmax, enhanced SUMOylation at K579 now produced a significant ∼37–70% increase in IA maximum conductance (Gmax) and a significant ∼30–50% increase in Kv4.2g surface expression that was accompanied by a 65% reduction in TC internalization. Blocking clathrin-mediated endocytosis (CME) in HEK cells expressing the Kv4.2 TC mimicked and occluded the effect of SUMO on IA Gmax; however, the amount of Kv4.2 associated with the major adaptor for constitutive CME, adaptor protein 2 (AP2), was not SUMO dependent. Thus, SUMOylation reduced Kv4.2 internalization by acting downstream of Kv4.2 recruitment into clathrin-coated pits. In sum, the two major findings of this study are: SUMOylation of Kv4.2 at K579 regulates TC internalization most likely by promoting channel recycling. Additionally, there is a reciprocity between Kv4.2 SUMOylation and the Kv4.2 interactome such that SUMOylation regulates the interactome and the interactome influences the pattern and effect of SUMOylation.

## Introduction

The transient potassium current (IA) is a rapidly inactivating subthreshold current that can be mediated by Kv4.1-3 α-subunits (Gutman et al., 2005). Kv4 channels contribute to IA in the heart (Niwa et al., 2008) and play a key role in shaping neuronal excitability, action potential firing rate, synaptic integration and plasticity (Jerng et al., 2004a). Dysregulation of Kv4 channels is associated with heart disease (Huo et al., 2014) as well as with neurological disorders, including epilepsy, Alzheimer’s disease, major depressive disorder, Huntington’s disease, and chronic pain (Hall et al., 2015; Scala et al., 2015; Kohling and Wolfart, 2016; Zemel et al., 2018; Carrillo-Reid et al., 2019; Noh et al., 2019; Aceto et al., 2020; Kanda et al., 2021; Kim et al., 2021; Wang et al., 2021).

Kv4 channels form a ternary complex (TC) with potassium channel interacting protein (KChIP1-4) and dipeptidyl peptidase-like protein (DPLP; DPP6 and 10). The TC is the fundamental unit that reproduces native IA in heterologous expression systems (Jerng et al., 2005; Amarillo et al., 2008; Jerng and Pfaffinger, 2012). Cytosolic KChIPs interact with the cytosolic N- and C-termini of Kv4 α-subunits at the ER to facilitate tetrameric assembly and efficient trafficking to the plasma membrane (Shibata et al., 2003; Kunjilwar et al., 2004). The transmembrane domain and the short intracellular N-terminus of DPLP interacts with Kv4 transmembrane domains 1 and 2 to promote exit from the ER (Ren et al., 2005; Cotella et al., 2012; Lin et al., 2014). Both DPLPs and KChIPs modulate channel gating at the plasma membrane (An et al., 2000; Bahring et al., 2001; Nadal et al., 2003; Shibata et al., 2003; Jerng et al., 2004b; Foeger et al., 2010). Dynamic interactions with several proteins beside KChIP and DPLP contribute to Kv4 assembly, trafficking, and function at the plasma membrane (Jerng and Pfaffinger, 2014). Tandem affinity purification of exogenously expressed Kv4.2 followed by mass spectrometry identified over 120 endogenous human embryonic kidney (HEK) cell proteins that associate with the α-subunit (Hu et al., 2020). These include proteins involved in channel folding and trafficking (Hasdemir et al., 2005; Chu et al., 2006; Yamakawa et al., 2007; Flowerdew and Burgoyne, 2009; Wang et al., 2012; Li et al., 2017; Hu et al., 2020; Tang, 2020), cytoskeletal and scaffolding proteins that organize the Kv4 macromolecular complex (Colledge et al., 2000; Petrecca et al., 2000; Wong et al., 2002; Gardoni et al., 2007; Lin et al., 2011), and ancillary proteins that influence channel properties (Kuryshev et al., 2000; Yang et al., 2001; Pourrier et al., 2003; Marionneau et al., 2012). The Kv4 macromolecular complex can also include other ion channels, such as Cav3.1-3 (Anderson et al., 2010a, b).

Limited progress has been made toward identifying the mechanisms regulating Kv4 protein–protein interactions (Morohashi et al., 2002; Cotella et al., 2012; Hu et al., 2020; Seifert et al., 2020). Expanding our knowledge of these mechanisms is key to understanding how IA is dynamically adapted. Post-translational SUMOylation regulates protein–protein interactions. Small ubiquitin like modifiers (SUMO1-5) are 11 kD peptides that can be conjugated to lysine (K) residues on target proteins (Flotho and Melchior, 2013; Celen and Sahin, 2020; Chang and Yeh, 2020; Henley et al., 2021). SUMO2 and SUMO3 are ∼97% identical and are referred to as SUMO2/3. SUMOylation is a dynamic modification, and the SUMOylation status of a target is determined by the opposing activities of the sole SUMO conjugating enzyme, Ubc9, and a variety of proteases that cleave SUMO from a target (Desterro et al., 1997). There are several non-mutually exclusive consequences of protein SUMOylation including: (1) promoting protein–protein interactions by linking the SUMO moiety on the target protein to a SUMO binding domain on the interacting partner (Psakhye and Jentsch, 2012; Seifert et al., 2015); (2) blocking protein–protein interactions, usually through steric hindrance (Dustrude et al., 2016); (3) altering post-translational decorations by competing with another post-translational K modification, such as ubiquitination (Anderson et al., 2012; Wang et al., 2014), and/or modulating phosphorylation at consensus sites containing a K (Hendriks et al., 2017); and, (4) interacting with phosphoinositides concentrated in the *trans*-Golgi (PI(3)P) and plasma membrane (PI(3,4,5)P_3_) (Kunadt et al., 2015).

Post-translational SUMOylation can regulate many processes in excitable tissue (Henley et al., 2021), and dysregulated SUMOylation can contribute to many disease states (Henley et al., 2018), including the nociceptor sensitization associated with chronic pain (Moutal et al., 2017; Wang et al., 2018; Forster et al., 2020; Jansen et al., 2021). SUMOylation can control ion channel transcription, translation, trafficking, surface expression, stability and biophysical properties (Rajan et al., 2005; Benson et al., 2007, 2017; Martin et al., 2007; Dai et al., 2009; Plant et al., 2011, 2012, 2020; Girach et al., 2013; Jaafari et al., 2013; Qi et al., 2014; Dustrude et al., 2016; Parker et al., 2017; Xiong et al., 2017; Yuan et al., 2017; Garcia-Caballero et al., 2019; Welch et al., 2019; Boulanger et al., 2021; Henley et al., 2021). We have previously shown that Kv4.2 channels can be decorated with SUMO2/3 in rat brain and in HEK cells (Welch et al., 2019). Software for predicting SUMOylation sites indicated that there were several putative SUMOylation sites on Kv4 α-subunits, including two sites that were conserved across isoforms and species, K437 and K579. Experimentally increasing baseline SUMOylation in HEK cells expressing a mouse Kv4.2 GFP fusion protein (Kv4.2g) increased channel SUMOylation only at K437 and K579 (Welch et al., 2019). This produced a significant 22–50% decrease in IA maximal conductance (Gmax) and a significant 70–95% increase in Kv4.2 surface expression. SUMOylation at K437 increased surface expression of electrically silent channels while SUMOylation at K579 mediated the significant decrease in IA Gmax without altering surface expression (Welch et al., 2019).

In our previous study, only Kv4.2g was over-expressed in HEK cells (Welch et al., 2019). HEK cells do not express DPLP and endogenous KChIP transcripts are nearly undetectable^1^. Since KChIP and DPLP can influence both the post-translational modification of Kv4 (Shibata et al., 2003; Seikel and Trimmer, 2009) and the effect of a given post-translational modification (Schrader et al., 2002; Prechtel et al., 2018), here we test if/how they uniquely influence Kv4.2 SUMOylation. In nociceptors, Kv4 channels exist in ternary complexes with KChIP isoforms 2 and 3 and DPP10 (Cheng et al., 2016; Kuo et al., 2017), here we study the effects of KChIP2a and DPP10c.

## Materials and Methods

### Chemicals and Antibodies

Chemicals were from Sigma unless stated otherwise. Antibodies are described in Table 1.

**TABLE 1:** Primary antibodies.

Antigen	Immunogen	Host species	Verified by	Manufacturer, catalog number	Concentration
GFP	Recombinant full-length protein corresponding to GFP	Rabbit, polyclonal	Specificity verified by company. On WB, the antibody recognizes recombinant GFP from HEK lysates.	Abcam, ab290	IP- 1 μL antibody per 0.5 mg protein. WB- 1: 20,000
Na^+^/K^+^- ATPase	Full length Rabbit alpha 1 Sodium Potassium ATPase	Mouse, monoclonal	Specificity verified by company. Positive signal on WB using HEK cell lysate	Abcam, ab7671	WB- 1:3000
Actin	Peptide mapped to C-terminus of human actin	Rabbit, polyclonal	Specificity verified by company. Positive signal on WB using C32 cell lysate	Santa Cruz Biotechnology, sc-1616-R	WB- 1:2000
BSA	Bovine serum albumin	Rabbit, polyclonal	Specificity verified by company. On WB, the antibody recognizes BSA protein.	Thermo Fisher, A11133	WB- 1:20,000
α-Adaptin	Mouse α-adaptin, amino acids 38-215	Mouse, monoclonal	Specificity verified by company using WB. siRNA against a-adaptin abolishes antibody signal (Zavodszky and Hegde, 2019). Antibody recognizes recombinant, purified AP2 complex on western blot. Protein recognized by antibody colocalizes with clathrin and other adaptor proteins (Umasankar et al., 2014).	BD Biosciences, 610502	WB- 1:2000

### Cell Culture

Human Embryonic Kidney 293 (HEK) cells were obtained from American Type Culture Collection (catalog number CRL-1573, lot number 2869494) and were cultured at 37°C and 5% CO_2_ in Eagle’s Minimum Essential Medium (MEM) (Corning, cat no. 10009CV) supplemented with 10% fetal bovine serum (ATCC, cat no. 30-2020) and 1% penicillin/streptomycin (Sigma, cat no. P4333). Cells were discarded after passage 20.

### Plasmids

A previously described plasmid containing the mouse Kv4.2 channel with GFP fused to the C-terminus (Kv4.2g) was provided by Dax Hoffman. The use of this plasmid is well established in the literature, and the GFP tag does not appear to disrupt Kv4 function or trafficking (Kim et al., 2007; Hammond et al., 2008; Lin et al., 2010). PCR-based site directed mutagenesis was performed as previously described to generate two mutations in the Kv4.2g plasmid, K437R and K579R (Welch et al., 2019). A plasmid containing SUMO-2 (Kamitani et al., 1998) was provided by Edward Yeh (Addgene plasmid #17360). A plasmid containing Ubc9 (Yasugi and Howley, 1996) was a gift from Peter Howley (Addgene plasmid #14438). pCMV6-KChIP2a (NM_145703) was purchased from Origene Technologies. PCR was used to add SalI and XhoI restriction sites at the beginning and end of the KChIP2a open reading frame (ORF) using the primers: SalI_KChIP2aFor: ATATATGTCGACGATGCGGGGCCAAGGCCGAAAGG and XhoI_KChIP2aRev: GAGTTTCTGCTCGAGCGGCCGCGTAC GCGTCTAGATGAC. The KChIP2a ORF was subcloned into pCMV-HA-N vector (Clontech) at the SalI and XhoI restriction sites to generate the vector pCMV-HA-N-KChIP2a. pCMV3-DPP10 (NM_199021) was purchased from Sino Biological. We note that this corresponds to the previously identified DPP10 isoform c (DPP10c) (Jerng et al., 2007). PCR was used to add SalI and XhoI restriction sites at the beginning and end of the DPP10c ORF using the primers: SalI_DPP10 For: ATATATGTCGACGATGACAGCCATGAAGC and XhoI_DPP10Rev: ATATATCTCGAGTTATTCATCTTCTTCT. The DPP10c ORF was subcloned into pCMV-HA-N vector (Clontech) at the SalI and XhoI restriction sites to generate the vector pCMV-HA-N-DPP10c.

### Transfection

Human embryonic kidney cells were transfected using the calcium phosphate transfection method. HEK cells were plated at 1.9 × 10^6^ cells/plate or 6 × 10^6^ cells/plate onto 60 or 100 mm plates, respectively, and incubated for 24 h. 10 or 25 μg of DNA were prepared in 250 or 440 μl TE buffer (10 mM Tris-HCl pH 8, 1 mM EDTA), and immediately before transfection 30 or 60 μl of 2 M CaCl_2_ followed by 250 or 500 μl 2X HBS (275 mM NaCl, 10 mM KCl, 12 mM dextrose, 1.4 mM Na_2_HPO_4_, 40 mM HEPES, and pH 7.05-7.1) was added dropwise to the DNA mixture, respectively. The transfection solution was added dropwise to the cells, and the cells were returned to 37°C/5% CO_2_ for 4 h followed by a media change. Equal amounts of each plasmid were used when co-transfecting multiple plasmids. Transfection efficiency was assessed 48 h later using fluorescence microscopy and only plates with >80% efficiency were used in experiments.

### Western Blot Assays

Proteins were resolved on 10% SDS-polyacrylamide gels and transferred for 1 h at 90 V to a PVDF membrane (Immobilon-P, cat# IPVH00010) using a wet electroblotting system (Bio-Rad, Mini *Trans*-Blot cell) and instructions provided by the manufacturer. Membranes were dipped in methanol and dried for 30 min. Membranes were blocked in 5% non-fat dry milk in TBS (50 mM Tris-HCl pH7.4, 150 mM NaCl) for 1 h at room temperature. Membranes were washed 1× with T-TBS (TBS + 0.1% Tween20) for 10 min. The membrane was incubated with primary antibodies (Table 1) in 1% non-fat dry milk in T-TBS overnight at 4°C with shaking. Membranes were washed 3× 5 min each with T-TBS. Membranes were incubated with the appropriate alkaline phosphatase conjugated secondary antibodies in 1% non-fat dry milk in T-TBS for 2 h at room temperature. Membranes were washed 3× with T-TBS for 10 min each. Alkaline phosphatase substrate was placed on the membrane for 5 min. Membranes were exposed to film (Research Products International) and chemiluminescent signals were visualized with a Kodak X-Omat 2000A imager. The optical density (OD) of a protein(s) of interest was measured using ImageJ software, as previously described (Parker et al., 2017; Welch et al., 2019).

In some cases, membranes were stripped and re-probed. In these cases, membranes were washed 2× in mild stripping buffer (20 mM glycine, 0.1% SDS, 1% Tween 20, 50 mM KCl, 20 mM Magnesium acetate, pH 2.2) for 10 min each. Membranes were then washed, 2× with PBS for 10 min each, and 2× with T-TBS for 5 min each. To ensure that the chemiluminescent signal was gone, alkaline phosphatase substrate was added to the membrane for 5 min and the membrane was exposed to film for 10 min. To account for technical variabilities such as differences in exposure time or loss of protein due to stripping, 0.1 or 0.2 μg BSA protein was always included in one lane on every gel and a primary antibody against BSA was always included for detection. The signal for the protein of interest on the pre- and post-stripped blot was always normalized by the BSA signal on the pre- and post-stripped blot, respectively.

In some cases, SYPRO Ruby Protein Blot Stain (Bio-Rad) was used to assess total protein. Whole cell lysates (10 μg) were resolved with PAGE and transferred to a PVDF membrane, as described above. Dried PVDF membranes were incubated protein side face-down in 7% acetic acid/10% methanol for 15 min and then washed 4×, 5 min each with dH_2_O. Membranes were placed protein side face-down in SYPRO Ruby Protein Blot Stain for 15 min and then washed 3×, 1 min each with dH_2_O. Stained protein was visualized using an Omega Ultra Lum 10gD imaging system using blue light transillumination. The optical density of the total protein signal in the entire lane was measured using ImageJ.

### Electrophysiology

For whole-cell patch clamp experiments, HEK cells were transfected with the appropriate DNA and incubated for 24 h. Cells were then passaged on to 100 mm coverslips coated with Poly-L-Lysine (50 μg/ml) and incubated. The next day, coverslips were transferred to a recording chamber and superfused continuously with extracellular saline (in mM: 141 NaCl, 4.7 KCl, 1.2 MgCl_2_, 1.8 CaCl_2_, 10 glucose, 10 HEPES, pH 7.4, and osmolarity ∼300 mOsm/L). Cells were visualized on an IX70 Olympus microscope, and transfected cells were identified with fluorescence microscopy. Fire polished borosilicate glass pipettes having a resistance of ∼2 MΩ were filled with intracellular saline (in mM: 140 KCl, 1 MgCl_2_, 1 CaCl_2_, 10 EGTA, 2 MgATP, 10 HEPES, pH 7.2, and osmolarity ∼290 mOsm/L) and connected to a MultiClamp 700A amplifier (Axon Instruments). After forming a GΩ seal, a slight negative pressure was used to break through the membrane, and only cells that maintained >700 MΩ seal following rupture and had less than 15 MΩ access resistance were examined. Whole cell capacitance was measured upon break-in. Fast and slow capacitance and series resistance were compensated. IA was elicited with a series of 1 s pre-pulses to −90 mV each followed by a 250 ms test-pulse ranging from −50 to +50 mV in 10 mV increments. The leak current from a pre-pulse to -30 mV was subtracted offline. Current density (peak current ÷ whole cell capacitance) is often used to compensate for IA variability introduced by cell size heterogeneity, thereby increasing statistical power. However, it was recently shown that this approach is only valid if there is a positive correlation between capacitance and current (Kula et al., 2020). We did not observe a positive correlation, so IA density was not examined. Instead, current (*I*) was converted to conductance (*G*) using the equation *G* = I/Vm − Vr, with Vm being the membrane potential and Vr being the reversal potential for potassium, −86 mV. The maximal conductance (Gmax), the voltage of half-activation (V50 act), and the slope of the activation curve were determined by plotting conductance against voltage and fitting the data with a first-order Boltzmann equation constrained to a zero minimum and extrapolated from −50 to −80 mV. The fast and slow time constants of inactivation (τfast and τslow) were determined by fitting the decay current for the +50 mV test-pulse with a two-term exponential equation. The voltage of half-inactivation (V50 inact) was measured with a series of 1.4 s pre-pulses from −110 to −30 mV in 10 mV increments, each followed by a 200 ms test pulse to +20 mV. Current was plotted against voltage and the data were fitted with a first-order Boltzmann equation extrapolated to −10 mV to determine V50 inact and the slope of the inactivation curve.

In some experiments, clathrin-mediated endocytosis was blocked with Pitstop2 (Abcam, ab120687). Cells were treated with 20 μM Pitstop2 for 20 min at 37°C/5% CO_2_ before transferring the coverslip to the recording chamber and Pitstop2 (20 μM) was included in the superfusate. Pitstop2 was prepared as a 30 mM stock in DMSO, where the working concentration of DMSO was 0.7%. Application of 0.7% DMSO for 20 min at 37°C/5% CO_2_ prior to transferring the cells to the recording chamber and inclusion of 0.7% DMSO in the superfusate did not affect IA Gmax (*n* = 3; Gmax, control: 87.44 nS vs. DMSO: 90.55 nS). In experiments using Pitstop2, enhanced SUMOylation was not achieved by co-transfecting SUMO and Ubc9; rather, SUMO2 and/or SUMO3 peptide (4.2 μM, Boston Biochem, #K-700) was dissolved in the intracellular saline in the patch pipette and delivered to the cell after whole-cell configuration was achieved. SUMO peptides were used at a concentration previously shown regulate the amplitude of kainate evoked current in HEK cells expressing GluK2 (Konopacki et al., 2011). We note that SUMO2 and SUMO3 are 97% identical and produced similar effects in this experiment. Both were employed because they were packaged together by the vendor.

### Cell Lysates to Measure Kv4.2 Expression

For experiments to measure steady-state Kv4.2g levels, transfected HEK cells on 100 mm culture dishes were washed 1× with ice-cold PBS and lysed in 1 ml RIPA buffer (1% NP40, 50 mM Tris-HCl pH7.4, 150 mM NaCl, 0.1% SDS, 0.5% DOC, 2 mM EDTA) supplemented with 20 mM *N*-Ethylmaleimide (NEM) and protease inhibitor cocktail (1:100, Sigma cat no. P8340) on ice for 30 min. Plates were scraped, and lysates were transferred to a 1.5 ml centrifuge tube. Cell debris was pelleted by centrifugation at 14,000 rpm for 15 min. Bicinchoninic acid (BCA) assay was performed to determine protein concentration, and whole cell lysate (10 μg of protein) was resolved with PAGE.

### Immunoprecipitation to Measure α-Adaptin

For experiments quantifying the amount of α-adaptin that co-immunoprecipitated (co-IP) with Kv4.2g, transfected HEK cells on 60 mm culture plates were washed 1× PBS. Cells were then lysed for 30 min on ice with IP/lysis buffer supplied with the Pierce Classic Magnetic IP/Co-IP kit (Thermo Fisher cat no. 88804) and supplemented with 20 mM NEM and protease inhibitor cocktail (1:100). Cells were scraped, transferred to a 1.5 ml centrifuge tube, and cell debris was pelleted for 10 min at 14,000 rpm. Protein concentration was determined with BCA, and 0.5 mg of protein was added to 1μl anti-GPF (Table 1). The lysate was incubated with the antibody at 4°C with shaking overnight, and IP was performed with the Pierce Magnetic Classic IP/Co-IP kit. IP was eluted in 50 μl elution buffer.

### Biotinylation of Cell Surface Proteins

NeutrAvidin resin (300 μL) (Thermo Fisher cat no. 29201) was placed in a Pierce Snap Cap Spin Column (Thermo Fisher cat no. 69725) and was equilibrated by washing 3× with PBS. Transfected HEK cells were washed 2× with PBS supplemented with 1.5 mM MgCl_2_ and 0.2 mM CaCl_2_ (PBS-CM) and incubated with EZ-Link Sulfo-NHS-SS-Biotin (1 mg/ml) (Thermo Fisher cat no. 21331) in PBS-CM for 30 min at 4°C. Unreacted biotin was quenched with PBS-CM +100 mM glycine at 4°C for 15 min, and cells were lysed for 30 min on ice in 500 μL RIPA buffer supplemented with protease inhibitor cocktail. Plates were scraped. The lysate was transferred to a clean 1.5 ml centrifuge tube. Cell debris was pelleted by centrifugation for 10 min at 14,000 rpm, and the supernatant was added to the washed NeutrAvidin resin and incubated for 2 h at 4°C with shaking. The column was placed in a clean 1.5 ml microcentrifuge tube and centrifuged for 2 min at 1,000 × *g*. The flow-through containing the unbiotinylated proteins was considered to be the intracellular fraction. The resin was washed 3× with Wash Buffer 1 (1% NP40, 1% SDS, and 1× PBS) and 3× with Wash Buffer 2 (0.1% NP40, 0.5 M NaCl, and 1× PBS). Biotinylated, cell surface proteins were eluted from the NeutrAvidin resin by incubating with 75 μl of 1× SDS buffer (50 mM Tris HCl pH 6.8, 2% SDS, 10% glycerol, 0.1% Bromophenol Blue, 100 mM DTT) with gentle shaking for 1 h at room temperature followed by centrifugation for 2 min at 1,000 × *g*. Intracellular and extracellular fractions along with 0.2 μg of BSA were used in western blot assays. Blots were cut horizontally at ∼50 kD. The lower portion of the blot was incubated with an antibody against actin. The upper portion of the blot was incubated with antibodies that recognized BSA and GFP. After measuring the OD for BSA and GFP, the blot was stripped and incubated with antibodies that recognize BSA and the Na^+^/K^+^-ATPase, whose expression did not change when SUMOylation was increased above baseline in HEK cells (Parker et al., 2017). The ODs of the extracellular GFP and Na^+^/K^+^-ATPase signals were normalized to their respective BSA signals, and then the normalized GFP signal was divided by the normalized Na^+^/K^+^-ATPase signal to determine Kv4.2 surface expression.

### Internalization Assay

Three plates of cells were used for each internalization experiment. One plate was used to measure internalization, one was used to measure total surface expression, and one was used to measure stripping efficiency. Cells were biotinylated and quenched as described above. The remainder of the experiment varied according to the plate.

Cells on the internalization plate were washed 1× in MEM. Fresh MEM was added, and the plate was incubated for 2.5 h at 18°C to allow for internalization with reduced degradation (Le et al., 1999). Cells were then washed 1× with NT buffer (150 mM NaCl, 1 mM EDTA, 0.2% BSA, and 20 mM Tris pH8.6). Surface-bound biotin was removed by incubating with 100 mM sodium 2-mercaptoethanesulfonate (MESNA) in NT buffer for 30 min at 4°C. Cells were washed 3× in NT buffer, 3× in PBS-CM and lysed with 500 μL RIPA buffer supplemented with protease inhibitor cocktail.

Immediately after the quenching step, cells on the strip plate and the total plate were washed 1× with NT buffer. Cells on the strip plate were incubated with MESNA in NT buffer for 30 min at 4°C to remove surface bound biotin. Cells on the total plate were incubated with NT buffer for 30 min at 4°C. Cells on the strip plate and the total plate were washed 3× in NT buffer, 3× in PBS-CM, and lysed with 500 μL RIPA buffer supplemented with protease inhibitor cocktail.

Protein concentration for each of the 3 lysates was determined with a BCA. For each lysate, 450 μg of protein was added to a Pierce Snap Cap Spin Column containing washed NeutrAvidin resin (300 μL) and incubated at 4°C overnight, shaking. The next day, the columns were placed in clean 1.5 ml microcentrifuge tubes, and the flowthroughs containing the unbiotinylated proteins were collected by centrifuging 2 min 1,000 × *g*. The resin was washed 3× with Wash Buffer 1 and 3× with Wash Buffer 2. Biotinylated proteins were eluted from the resin with 75 μl 1× SDS buffer by shaking for 1 h at room temperature. Biotinylated fractions were collected by centrifuging at 1,000 × *g* for 2 min.

The unbiotinylated and biotinylated fractions from the three plates were resolved with PAGE. Blots were probed with anti-GFP. The percentage of Kv4.2g internalized was calculated as the optical density (OD) of the biotinylated Kv4.2g signal from the internalized plate divided by the OD of the biotinylated Kv4.2g signal from the total surface expression plate multiplied by 100. The stripping efficiency was calculated as the OD of the biotinylated Kv4.2g signal from the strip plate divided by the OD of the biotinylated Kv4.2g signal from the total surface expression plate, subtracted from 1 and multiplied by 100. Only data from experiments where the stripping efficiency was >90% were included.

### Statistical Analysis

GraphPad PRISM 9 software was used for statistical analysis of data. Normality and homogeneity of variance was assessed for each data set. Data points >2 standard deviations from the mean were considered outliers and were excluded. This resulted in removal of 1 data point from each treatment group. In all cases, the significance threshold was set at *p* < 0.05. Data were analyzed with t-tests or one-way ANOVAs with Tukey *post hoc* tests that made all pairwise comparisons. Each statistical test performed, and the result of that test are listed in each Figure legend.

## Results

### SUMOylation of Kv4.2g at K579 Increases IA Gmax in the Presence of Auxiliary Subunits, KChIP2a and DPP10c

Previous studies showed that Kv4, KChIP and DPLP proteins will interact to form a TC in native cells and when overexpressed in heterologous model systems (Seikel and Trimmer, 2009; Jerng and Pfaffinger, 2014), including HEK cells (Foeger et al., 2012; Lainez et al., 2018; Murphy and Hoffman, 2019; Zhang et al., 2020). In the experiments reported here, HEK cells, which do not endogenously express Kv4 channels, were transiently transfected with Kv4.2g and the hemagglutinin (HA)-tagged auxiliary subunits KChIP2a (HA-KChIP2a) and DPP10c (HA-DPP10c). Consistent with previous observations (Murphy and Hoffman, 2019), relative to HEK cells expressing Kv4.2g alone, co-expression of KChIP2a and DPP10c produced a significant ∼2-fold increase in IA maximal conductance (Gmax) (Supplementary Figure 1A), as well as a significant hyperpolarization of the voltage of half activation (V50 act) and a significant depolarization of the voltage of half inactivation (V50 inact) (Supplementary Figure 1B). As expected (Jerng et al., 2007), measures of the half inactivation time (time at which half of the peak current is inactivated) showed that channel inactivation was accelerated when Kv4.2 was incorporated into the ternary complex (Supplementary Figure 1C). At positive voltages, the rate of decay was constant, but it increasingly slowed as voltages became more negative. This contrasts with oocyte expression of a TC comprising Kv4.2 + DPP10c + KChIP3, which showed a fairly constant rate of decay at negative voltages and a gradual slowing with increasing positive voltages (Jerng et al., 2007). The biophysical properties of Kv4 complexes depend upon their exact subunit composition. Different isoforms of DPLP and KChIP will produce TCs with distinct biophysical phenotypes. Our data highlight the limitations of characterizing only one combination of subunits, and we note that our findings for a TC comprising Kv4.2 + KChIP2a + DPP10c may not apply to all Kv4 TCs. Preliminary immunoprecipitation (IP) experiments also confirmed that Kv4.2g non-covalently bound KChIP2a and DPP10c in co-transfected HEK cells (Supplementary Figure 1D).

**FIGURE 1:**
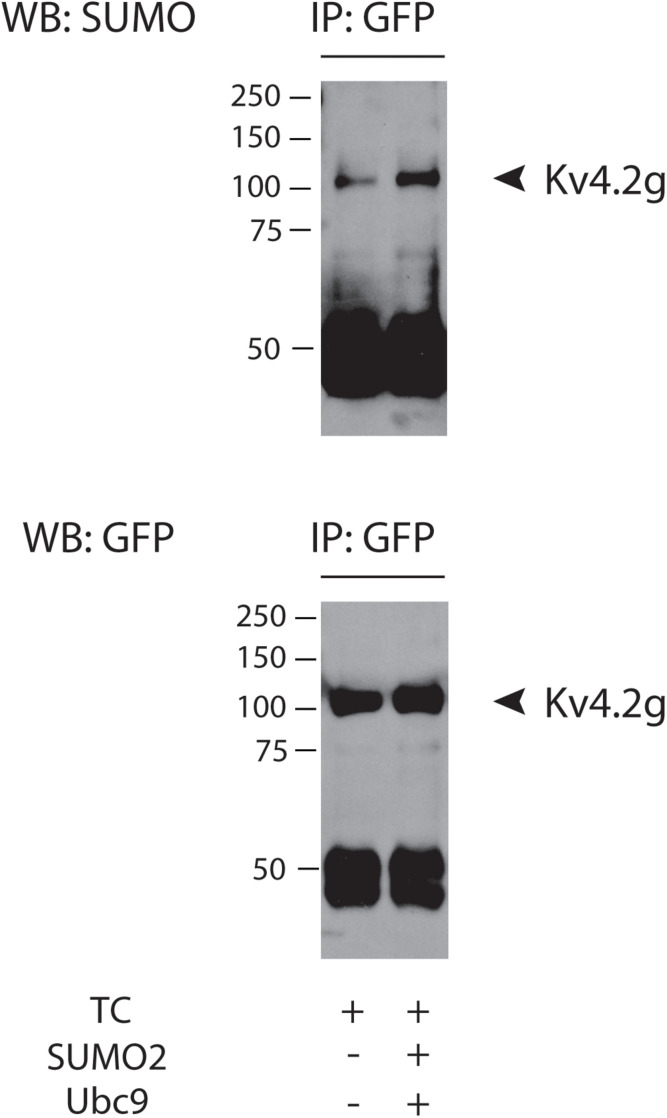
Kv4.2g is SUMOylated in the TC. Kv4.2g was co-expressed with HA-KChIP2a and HA-DPP10c in HEK cells with (+) or without (–) co-transfection of SUMO2 + Ubc9. Anti-GFP IPs were followed by western blotting. Blots were probed with anti-SUMO, and then stripped and reprobed with anti-GFP. Note the band at ∼50 kD represents the antibody used in the IP.

We next sought to determine if Kv4.2g could be decorated with SUMO when it was incorporated into the ternary complex. SUMOylation of target proteins can be increased by globally augmenting the concentration of SUMO and the SUMO conjugating enzyme, Ubc9, in tissue culture cells (Dai et al., 2009; Parker et al., 2017; Welch et al., 2019). In these experiments, SUMOylation was enhanced by co-expressing two plasmids, one that encoded SUMO2 and one that encoded Ubc9. Note that SUMO2 was chosen because we previously demonstrated that Kv4.2 was decorated with SUMO2/3 but not SUMO1 in rat brain membrane preparations (Welch et al., 2019). HEK cells were transiently transfected with plasmids encoding Kv4.2g, HA-KChIP2a, and HA-DPP10c with or without co-transfection of plasmids encoding SUMO2 and Ubc9. Kv4.2 was isolated from cell lysates using immunoprecipitation with an antibody that recognizes GFP (anti-GFP). IP products were used to generate western blots. The blots were first probed with an antibody that recognizes SUMO2/3 (anti-SUMO), then they were stripped and re-probed with anti-GFP. The data indicated that Kv4.2g showed baseline SUMOylation when incorporated into the ternary complex, and globally enhancing SUMOylation increased the number of SUMO2 decorations on Kv4.2g (Figure 1).

**FIGURE 2:**
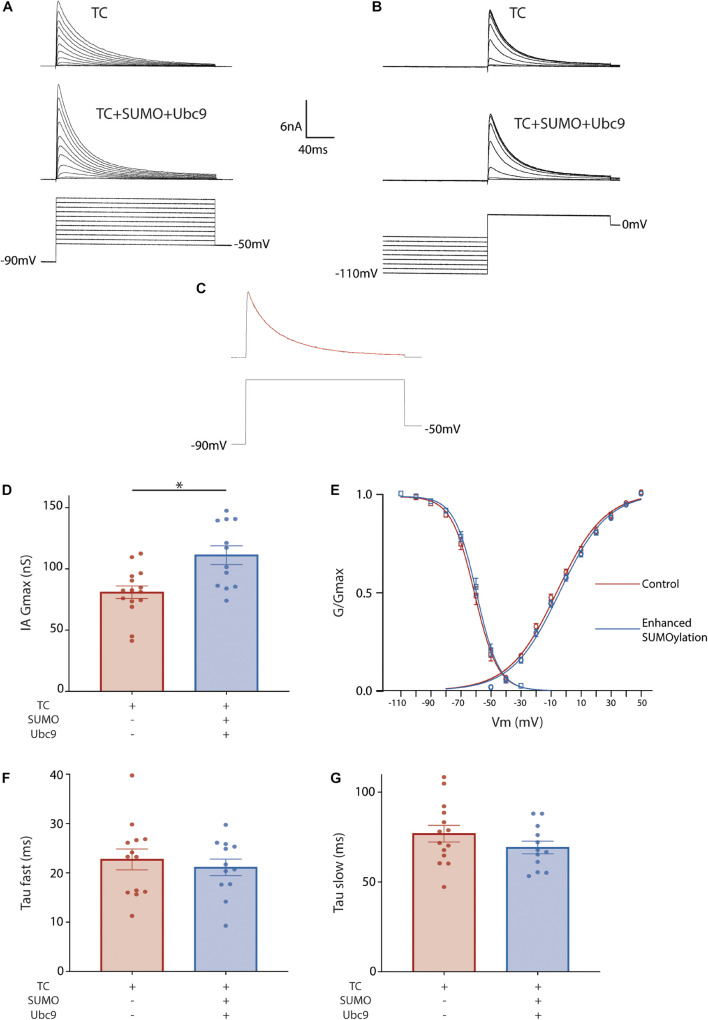
Enhanced SUMOylation increases IA Gmax mediated by the TC. Whole cell-patch clamp recordings for HEK cells transiently transfected with Kv4.2g + HA-KChIP2a + HA-DPP10c. SUMOylation was (+) or was not (–) enhanced by co-expressing SUMO2 + Ubc9. **(A)** Representative current traces (upper) and voltage protocol (lower) for obtaining the voltage dependence of activation. **(B)** Representative current traces and voltage protocol used for obtaining the voltage dependence of steady-state inactivation. **(C)** Representative curve fit used for obtaining the fast and slow time constants of inactivation. **(D)** Plots of mean ± SEM IA Gmax. Each dot represents one cell from ≥3 transfections. Asterisks indicate significant differences. TC, 80.9 ± 5.1 nS vs. TC + SUMO2 + Ubc9, 111.2 ± 7.6 nS; *t*-test *p* = 0.002. **(E)** Activation (circle) and steady-state inactivation (square) curves. The points on the curve represent the mean ± SEM for cells shown in **(D)**. TC V50 Act, –5.8±1.2 mV, *n* = 15; TC + SUMO2 + Ubc9 V50 Act, –2.5 ± 1.4 mV *n* = 12; *t*-test, *p* = 0.09. TC V50 Inact, –60.8 ± 1.4 mV, *n* = 16; TC + SUMO2 + Ubc9 V50 Inact, –58.7 ± 1.5 mV, *n* = 11; *t*-test, *p* = 0.32. Error bars are plotted for all data points. Plots of the mean ± SEM fast **(F)** and slow **(G)** time constants of inactivation. TC τf, 21.4 ± 1.5 ms; TC + SUMO2 + Ubc9 τf, 21.1 ± 1.7 ms; *t*-test *p* = 0.8871; TC τs, 78.9 ± 5.5 ms; TC + SUMO2 + Ubc9 τs, 69.2 ± 3.5 ms; *t*-test *p* = 0.1415.

To investigate if/how SUMO modulates IA mediated by the Kv4.2g TC, IA was characterized with whole cell patch clamp in cells expressing Kv4.2g + HA-KChIP2a + HA-DPP10c with or without SUMO2 + Ubc9. The voltage dependence of activation was determined with a series of depolarizing test pulses from −50 to 50 mV, each preceded by a hyperpolarizing prepulse to −90 mV (Figure 2A). The voltage dependence of steady-state inactivation was examined with a series of hyperpolarizing prepulses from −110 to −30 mV, each followed by a depolarizing testpulse to 20 mV (Figure 2B). The time constants for inactivation were determined by eliciting a current with a hyperpolarizing prepulse to −90 mV followed by a depolarizing testpulse to 50 mV, and then fitting the decay with a double exponential equation (Figure 2C). There was a significant 37% increase in IA Gmax when SUMO and Ubc9 were co-expressed with the Kv4.2g ternary complex relative to cells only expressing the Kv4.2g ternary complex (Figure 2D). SUMO and Ubc9 co-expression had no significant effect on the V50 act, the V50 inact, the slopes of the activation and inactivation curves, or the fast (τ fast) and slow (τ slow) time constants of inactivation (Figures 2E–G).

**FIGURE 3:**
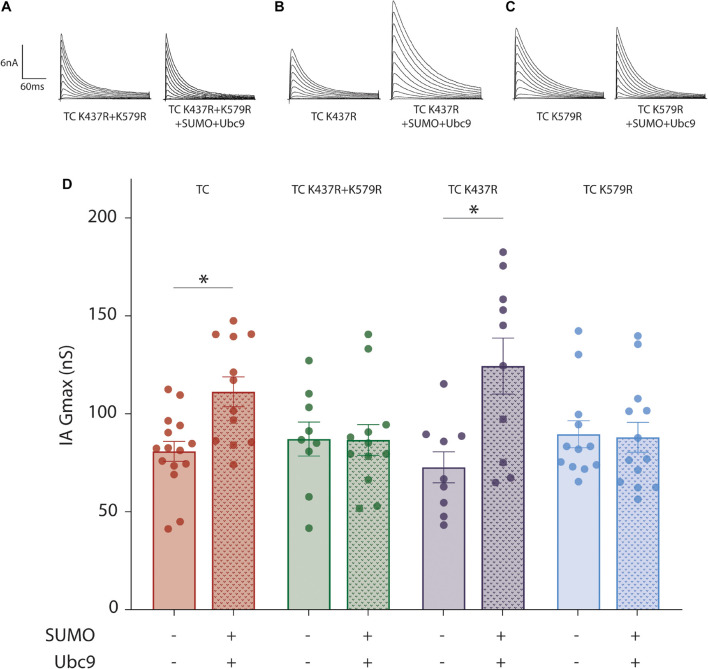
SUMOylation of Kv4.2g at K579 increases IA Gmax mediated by the TC. A mutant Kv4.2 plasmid, K437R + K579R or K437R or K579R, was co-expressed with wild type HA-KChIP2a and HA-DPP10c in HEK cells to generate mutant TCs. **(A–C)** Representative current traces for mutant TCs when SUMOylation was or was not increased by co-transfecting SUMO2 + Ubc9. **(D)** Bar graph showing the mean ± SEM IA Gmax for each treatment group. Note that wild-type data are replotted from Figure 1 for the sake of comparison. Each dot represents one cell collected from ≥3 transfections. Asterisk, significant differences. TC K437R + K579R: control, 87.11 ± 8.7 nS vs. SUMO2 + Ubc9, 86.7 ± 7.8 nS, *t*-test *p* = 0.97; TC K437R: control, 72.7 ± 7.9 nS vs. SUMO2 + Ubc9, 124.4 ± 14.3 nS, *t*-test *p* = 0.007; TC K579R: control, 89.6 ± 6.9 nS vs. SUMO2 + Ubc9, 88.0 ± 7.6 nS, Mann–Whitney *U p* = 0.72.

The increase in IA Gmax could potentially be due to SUMOylation of Kv4.2g, HA-KChIP2a and/or HA-DPP10c. We first tested if SUMOylation of Kv4.2g at K437 and/or K579 was necessary for the significant increase in IA Gmax by repeating the patch-clamp experiments with three Kv4.2g mutants: K437R, K579R, and K437R + K579R (Figure 3). Incorporating the Kv4.2g double mutant into the TC abolished the SUMOylation-induced increase in IA Gmax, suggesting that SUMOylation of Kv4.2g was necessary for the enhancement. Augmenting SUMOylation in HEK cells expressing Kv4.2g K437R still produced a significant increase in IA Gmax, but not when the TC contained Kv4.2g K579R. These data suggested that SUMOylation of Kv4.2g K579 produced a significant increase in the current mediated by the TC. We next examined the mechanisms involved.

### SUMOylation of Kv4.2g K579 Does Not Enhance Kv4.2 Protein Expression

**FIGURE 4:**
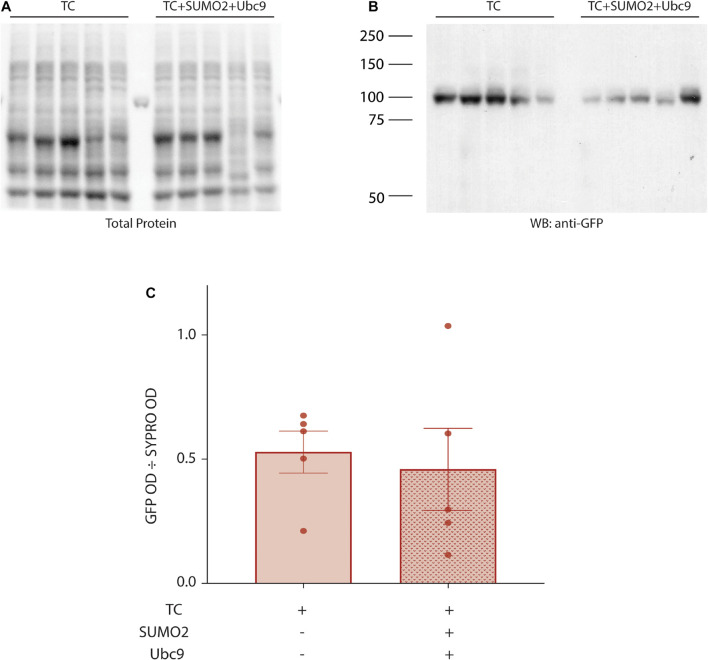
Kv4.2g steady-state levels are not altered by enhancing SUMOylation. Lysates (10 μg) from HEK cells expressing Kv4.2g+ HA-KChIP2a+ HA-DPP10c with (+) or without (–) SUMO2 + Ubc9 were resolved with PAGE and transferred to a PVDF membrane. Total protein was visualized with SYPRO Ruby blot stain. Afterward, the same membrane was probed with anti-GFP to visualize Kv4.2g. Kv4.2g protein expression was quantified by dividing the OD for the Kv4.2g signal by the OD for the total protein signal in the entire lane. Western blot showing **(A)** SYPRO Ruby total protein staining and **(B)** Kv4.2g signal. Each lane represents one independent experiment. The source of the variability between experiments is not known. Note that the single band in the lane between TC and TC + SUMO + Ubc9 on the SYPRO Ruby stained blot is one rung on the pre-stained molecular weight ladder. **(C)** Bar graph showing mean ± SEM Kv4.2g protein expression. Each data point represents one independent experiment. TC, 0.53 ± 0.19 vs. TC + SUMO2 + Ubc9, 0.46 ± 0.37; *t*-test *p* = 0.718.

In these experiments, SUMOylation is augmented by co-expressing SUMO2 and Ubc9, and this could potentially enhance IA Gmax by increasing Kv4.2g protein expression. We tested this idea by transiently transfecting HEK cells with Kv4.2g + HA-KChIP2a + HA-DPP10c with or without transient transfection of SUMO2 + Ubc9. Cell lysates were used in western blot experiments (Figure 4). Total protein in a cell lysate was first estimated by staining the blot with SYPRO Ruby and integrating the signal over an entire lane, as described in Section “Materials and Methods” (Figure 4A). Note Kv4.2 expression is not high enough to produce a distinct band with this method of visualization. Afterward, Kv4.2g protein on the same western blot was visualized with anti-GFP (Figure 4B). The amount of Kv4.2g protein was defined as the optical density (OD) for the anti-GFP signal divided by the OD for SYPRO Ruby stain for the same lysate (Figure 4C). There was no significant difference in Kv4.2g protein expression between the two treatment groups (Figure 4C). These data indicate that Kv4.2g SUMOylation did not increase IA Gmax by increasing Kv4.2g protein expression.

### SUMOylation of Kv4.2g at K579 Increases TC Surface Expression by Reducing Internalization

**FIGURE 5:**
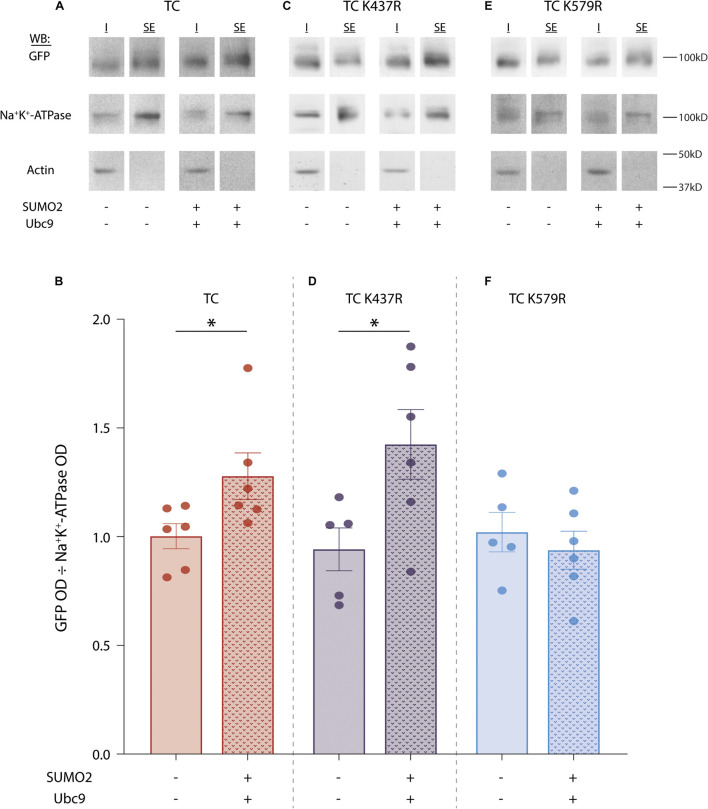
SUMOylation of Kv4.2g at K579 increases surface expression. SUMOylation was (+) or was not (–) increased in HEK cells expressing wild-type TC, TC K437R, or TC K579R. Surface proteins were biotinylated, cells were lysed, and surface expressed fractions (SE) were isolated using NeutrAvidin resin. Proteins that did not bind to the NeutrAvidin resin were considered to be the intracellular fraction (I). **(A,C,E)** Western blots containing SE and I from one representative experiment for each treatment group. The upper portion of the blot (above 50 kD) was used to visualize Kv4.2g with anti-GFP; it was then stripped and re-probed with anti-Na^+^/K^+^-ATPase. The lower portion of the blot (below 50 kD) was probed for actin. **(B,D,F)** Bar graphs showing the mean ± SEM Kv4.2g surface expression [SE Kv4.2g OD/SE Na^+^/K^+^-ATPase OD]. Each dot is one independent experiment. Asterisk, significantly different. TC: control, 1.0 ± 0.1 vs. SUMO2 + Ubc9, 1.3 ± 0.1, *t*-test *p* = 0.046; TC K437R: control, 0.9 ± 0.1 vs. SUMO2 + Ubc9, 1.4 ± 0.2, *t*-test *p* = 0.037; TC K579R: control, 1.0 ± 0.1 vs. SUMO2 + Ubc9, 0.9 ± 0.1, *t*-test *p* = 0.528.

Since SUMOylation did not increase Kv4.2g protein expression, we next asked whether the significant increase in IA Gmax was mediated by an increase in Kv4.2g surface expression. To do this, we performed cell surface biotinylation assays on HEK cells expressing the TC components with or without co-transfection of SUMO2 + Ubc9. Surface proteins were biotinylated, cells were lysed, and the surface fraction was isolated using NeutrAvidin resin. Proteins that did not bind to the NeutrAvidin resin were considered to be intracellular. Intracellular and surface fractions from each treatment group were used in western blot experiments (Figure 5A). Blots were cut horizontally at ∼50 kD and the upper portion of the blot was probed for Kv4.2g. After obtaining the OD for the Kv4.2g signal, the blot was stripped and re-probed for another cell surface protein, Na^+^K^+^-ATPase, whose expression is not altered by increasing SUMOylation in HEK cells (Parker et al., 2017). The lower portion of the blot was probed for actin to ensure there was no intracellular contamination in the surface fraction. Kv4.2g surface expression was defined as the Kv4.2g OD in the surface fraction divided by the Na^+^K^+^-ATPase OD in the surface fraction. A plot of the data indicated that Kv4.2g surface expression increased by 30% when SUMOylation was enhanced (Figure 5B). This is consistent with the SUMOylation-induced ∼37% increase in IA Gmax (Figure 2).

The experiment was repeated using the mutants, Kv4.2g K437R (Figures 5C,D) or Kv4.2g K579R (Figures 5E,F). Enhanced SUMOylation still elicited an increase in surface expression when the TC comprised Kv4.2g K437R (Figure 5D) but not Kv4.2g K579R (Figure 5F). These data indicated that SUMOylation of the TC at Kv4.2 K579 increased channel surface expression and IA Gmax.

**FIGURE 6:**
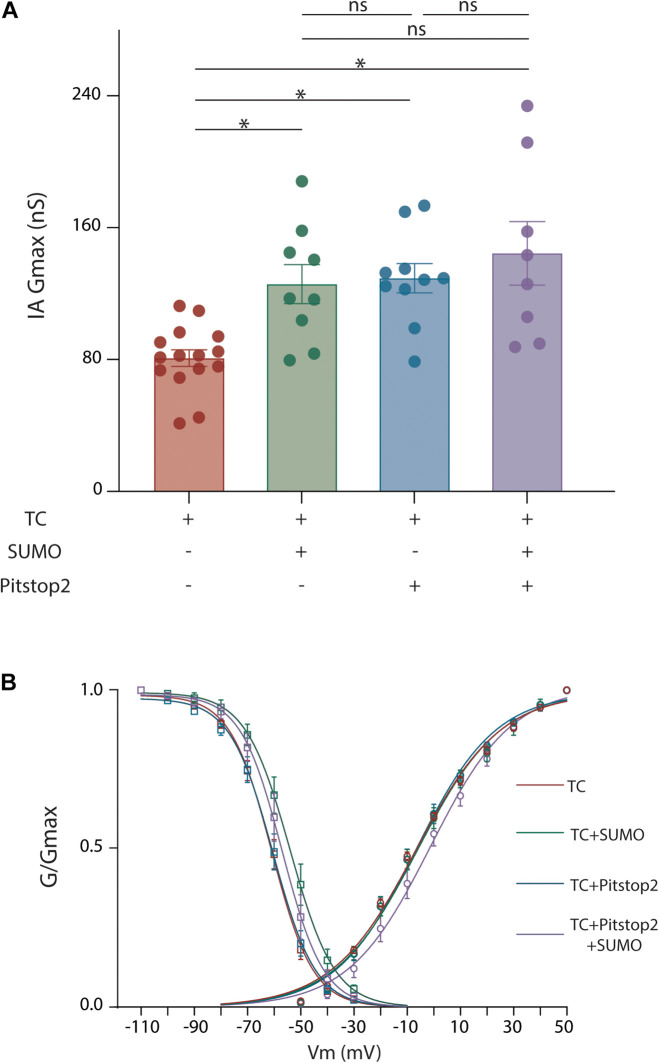
Blocking endocytosis mimics and occludes the effects of Kv4.2 K579 SUMOylation. HEK cells were transiently transfected with plasmids encoding the three TC components. Whole cell patch clamp recordings were performed on transfected cells with and without SUMO(2 or 3) peptides in the patch pipette and Pitstop2, a cell permeable endocytic inhibitor, in the superfusate. **(A)** Plot showing the mean ± SEM IA Gmax for each treatment group. Asterisks indicate significance. One-Way ANOVA with Tukey’s *post hoc*, *F*(3,38) = 8.013, *p* = 0.0003; TC: 80.87 ± 5.1 nS, TC + SUMO: 125.8 ± 11.8 nS, TC + Pitstop2: 129.4 ± 8.9 nS, TC + Pitstop2 + SUMO: 144.4 ± 19.2 nS. **(B)** Activation (circle) and steady-state inactivation (square) curves for each treatment group. Each data point represents the mean ± SEM for all data points analyzed. There was no significant difference in the voltage dependence across treatment groups: V50 act, *F*(3,39) = 0.358, *p* = 0.784; V50 inact, *F*(3,42) = 2.70, *p* = 0.0578.

Membrane protein SUMOylation is known to modulate a protein’s surface expression by regulating both anterograde transport from the Golgi to the plasma membrane (Zhou et al., 2018) and internalization from the plasma membrane (Dustrude et al., 2016; Ma et al., 2016). We first tested if SUMOylation reduced internalization. Current data suggest that Kv4.2 undergoes clathrin-mediated endocytosis (CME) (Kim et al., 2007; Nestor and Hoffman, 2012; Hu et al., 2020). We therefore performed patch clamp recordings to measure IA when CME was inhibited with Pitstop2, a cell-permeable clathrin inhibitor. Application of Pitstop2 (20 μm, 20 min) to HEK cells expressing Kv4.2g + KChIP2a + DPP10c produced a significant 59% increase in IA Gmax compared to control (Figure 6A and Table 2). To test if Pitstop2 mimicked and occluded the effects of SUMOylation, SUMO2 or SUMO3 peptide was acutely delivered to a HEK cell after endocytosis was inhibited. To do this, Pitstop2 was or was not pre-applied for 20 min followed by whole-cell patch clamp with the SUMO peptide included in the recording pipette (4.2 μM). This contrasts with all other experiments where an increase in SUMOylation was achieved by transient expression of plasmids encoding SUMO2 and Ubc9. Treatments with SUMO2/3 or Pitstop2 or SUMO2/3 + Pitstop2 produced a similar increase in IA Gmax relative to untreated controls; however, IA Gmax was not significantly different between these three treatment groups (Figure 6 and Table 2). Thus, Pitstop2 mimicked and occluded the effect of SUMO2/3. Because the effects of SUMO2/3 and Pitstop were not additive, these data support the hypothesis that SUMOylation increased channel surface expression and IA Gmax largely by reducing internalization, rather than by increasing anterograde transport. It should be noted that the level of internalization depends upon the rates of two opposing processes: endocytosis and recycling from the endosome back to the plasma membrane. Recycling is downstream of endocytosis, and the amount of recycled biotinylated Kv4 will depend upon the amount of internalized biotinylated Kv4. Since Pitstop2 blocks endocytosis, it will also affect recycling, and these data do not indicate if SUMO regulates endocytosis and/or recycling.

**TABLE 2:** Whole-cell patch clamp data when clathrin-mediated endocytosis was blocked using Pitstop2.

	TC	TC+ Pitstop2	TC+ SUMO	TC+ Pitstop2+ SUMO
Gmax (nS)	80.87 ± 5.1	129.4 ± 8.9^T^	125.8 ± 11.8^T^	144.4 ± 19.2^T^
V50 act (mV)	−5.8 ± 1.2	−5.3 ± 2.7	−5.3 ± 2.0	−2.7 ± 3.1
Slope act	15.91 ± 0.26	14.6 ± 0.69	15.44 ± 0.55	14.45 ± 0.47
V50 inact (mV)	−60.82 ± 1.4	−60.37 ± 1.8	−54.18 ± 1.9	−56.75 ± 2.5
Slope inact	−6.901 ± 0.30	−6.913 ± 0.24	−7.772 ± 0.50	−6.489 ± 0.28
τ fast (ms)	22.72 ± 2.1	19.66 ± 2.2	23.13 ± 3.4	20.32 ± 2.4
τ slow (ms)	76.89 ± 4.6	92.10 ± 18.9	94.94 ± 15.6	79.70 ± 4.3

*TC is the ternary complex and includes Kv4.2g, HA-KChIP2a, and HA-DPP10c. Means ± SEM are listed.*

*^T^Significantly different from Kv4.2g + HA-KChIP2a + HA-DPP10c. One-way ANOVA with Tukey’s multiple comparisons test, F(3,38) = 8.013, p = 0.0003.*

*V50 act is not significantly different: F(3,39) = 0.358, p = 0.784.*

*V50 inact is not significantly different: F(3,42) = 2.70, p = 0.0578.*

**FIGURE 7:**
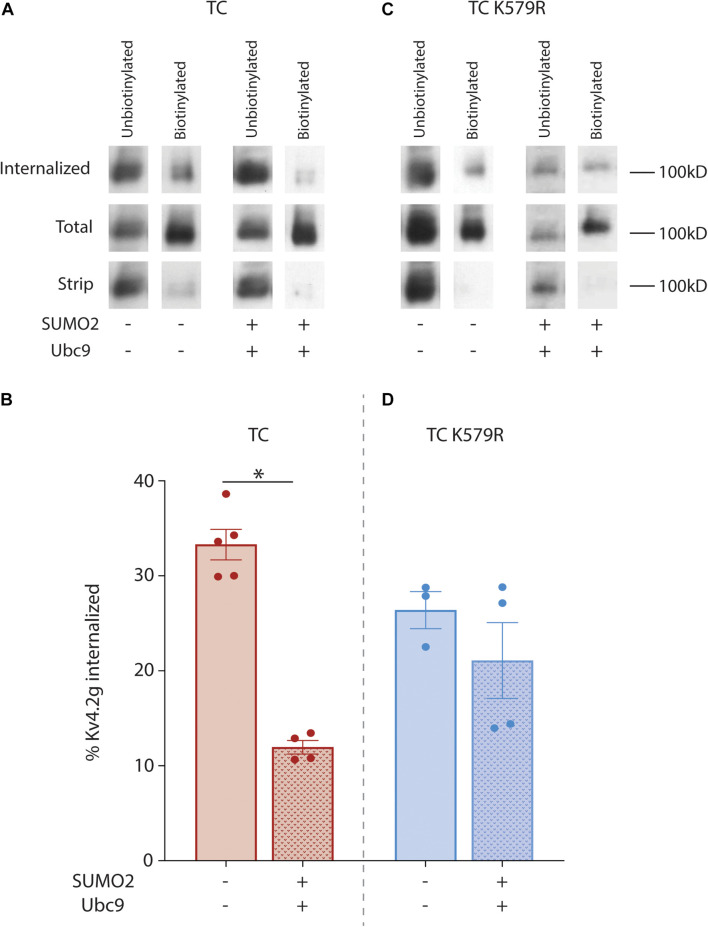
SUMOylation of Kv4.2g at K579 reduces channel internalization. HEK cells were co-transfected with plasmids encoding HA-KChIP2a+HA-DPP10c with either Kv4.2g or Kv4.2g K579R. SUMOylation was (+) or was not (–) enhanced by co-expressing SUMO2 and Ubc9. Assays to measure internalization in the two treatment groups were performed as detailed in the text. **(A,C)** Western blots containing the unbiotinylated and biotinylated fractions from the internalized, total, and strip plate lysates for a representative experiment in each treatment group for wild-type and mutant TCs. Blots were probed with anti-GFP to visualize Kv4.2g α-subunits. **(B,D)** Bar graph representing the mean ± SEM percent internalization: (OD internalized, biotinylated fraction ÷ OD total, biotinylated fraction) × 100. The data for an experiment was only included if the stripping efficiency was >90%: [1-(OD strip, biotinylated fraction ÷ OD total, biotinylated fraction)] × 100. Each data point represents one independent experiment. Asterisk, significantly different. TC: control, 33.3 ± 1.7% vs. SUMOs + Ubc9, 12 ± 0.7%, *t*-test *p* = <0.0001; TC K579R: control, 26.4 ± 1.2% vs. SUMO2 + Ubc9, 21.1 ± 4%, *t*-test *p* = 0.337.

In order to further test whether SUMOylation of Kv4.2 was necessary to reduce TC internalization, Kv4.2 internalization was analyzed with a biotin internalization assay. The assay was performed on two treatment groups: HEK cells expressing the Kv4.2g TC or the Kv4.2g TC + SUMO + Ubc9. Three plates of cells were used for one experiment in one treatment group. The 1st plate was used to measure internalized channels. Cells were treated with biotin on ice to label surface proteins and prevent endocytosis. Labeled cells were then placed at 18°C for 2.5 h to allow for internalization with reduced degradation (Le et al., 1999). Upon completion of the incubation, cells were treated with MESNA to cleave biotin from surface proteins. Biotinylated Kv4.2g channels that were internalized during the 18°C internalization step were protected from cleavage by MESNA, and following cell lysis, internalized biotinylated proteins were isolated using NeutrAvidin resin. The 2nd plate was used to measure total Kv4.2g surface expression. Immediately following biotinylation on ice, cells on this plate were lysed and biotinylated proteins were isolated using NeutrAvidin resin. Since there was no incubation at an elevated temperature, biotinylated proteins should largely represent total surface expression. The 3rd plate served as a control to determine stripping efficiency. Immediately following biotinylation on ice, cells on this plate were treated with MESNA to remove all biotin from surface expressed proteins. NeutrAvidin resin was once again used to isolate any remaining biotinylated proteins. The unbiotinylated and biotinylated fractions from the three plates were resolved with PAGE followed by western blot experiments using anti-GFP to visualize Kv4.2g (Figure 7A). MESNA stripping efficiency was determined by dividing the OD for the biotinylated Kv4.2g signal on the stripped plate (3rd plate) by the OD for the biotinylated Kv4.2g signal on the total plate (2nd plate), subtracting from 1 and multiplying by 100. An experiment was not used if the stripping efficiency was <90% (11% of experiments). If stripping efficiency was >90%, then the percentage of internalized Kv4.2g channels was determined by dividing the OD for the biotinylated Kv4.2g signal on the internalization plate (1st plate) by the OD for the biotinylated Kv4.2g signal on the total plate (2nd plate) and multiplying by 100. Plotting the data for both treatment groups showed that enhancing SUMOylation significantly decreased Kv4.2g internalization by 64% relative to control (Figure 7B). We have shown SUMOylation of K579 but not K437 is necessary for an increase in Kv4.2g surface expression (Figures 3, 5). In order to determine if SUMOylation of Kv4.2g K579 was necessary for the SUMOylation induced decrease in Kv4.2g internalization, these experiments were repeated using the Kv4.2g K579R mutant (Figure 7C). Plotting the data for the two treatment groups showed that enhancing SUMOylation had no significant effect on internalization when K579 was mutated to R (Figure 7D). In addition, the mutation alone significantly reduced internalization by ∼20% (% internalized: TC, 33.3% ± 1.6 vs. TC K579R, 26.4% ± 2.0; *p* = 0.037, *t*-test). Together these data suggest that K579 influences internalization, and post-translationally decorating K579 with SUMO significantly reduces internalization.

### The Effect of K579 SUMOylation on Kv4.2g Internalization Is Downstream of Cargo Recruitment and Vesicle Formation

**FIGURE 8:**
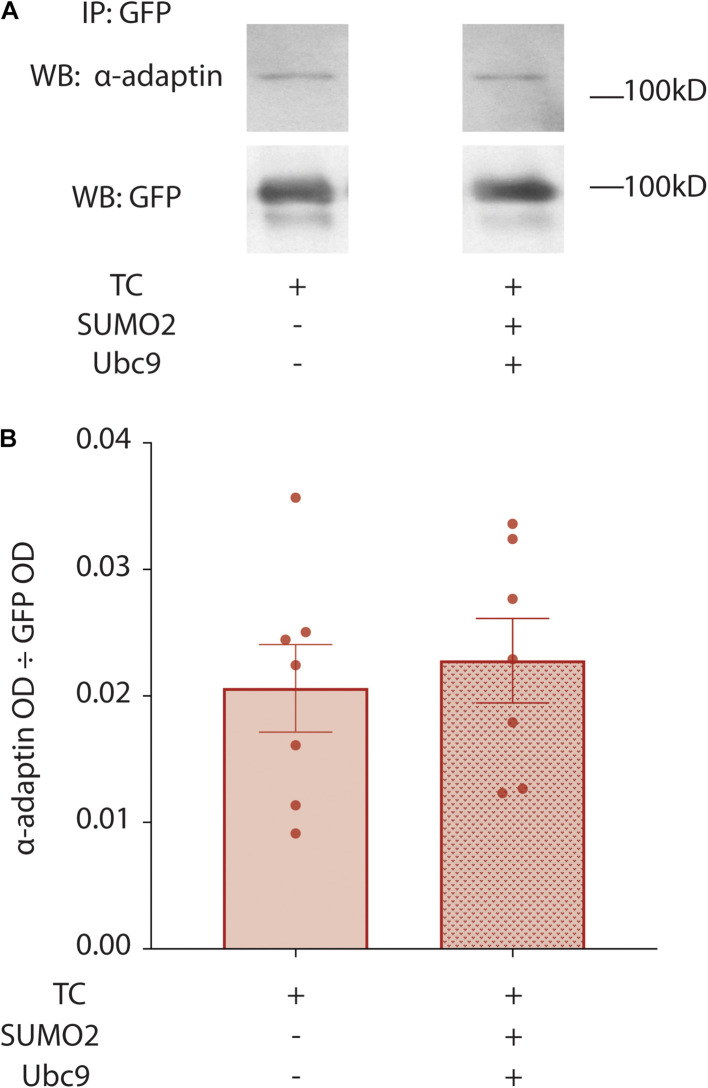
Enhancing SUMOylation does not change the fraction of Kv4.2g associated with α-adaptin. HEK cells were co-transfected with the TC components, and SUMOylation was (+) or was not (–) increased by co-expressing SUMO2 and Ubc9. Kv4.2g IP was obtained from cell lysates using an anti-GFP antibody, and IP products were resolved with PAGE. Western blots were probed for α-adaptin and then stripped and re-probed for Kv4.2g. **(A)** Representative western blots for α-adaptin and Kv4.2g for control and enhanced SUMOylation treatment groups. **(B)** Bar graphs plotting the mean ± SEM of α-adaptin that co-IPs with Kv4.2g for each treatment group [OD α-adaptin ÷ OD Kv4.2g]. Each data point is one independent experiment. TC, 0.02 ± 0.0035 vs. TC + SUMO2 + Ubc9, 0.02 ± 0.0033; *t*-test *p* = 0.657.

Internalization of Kv4.2g in the previous experiments was largely determined by the rates of two opposing processes: endocytosis and recycling of the endocytosed channel from the endosome back to the plasma membrane. Note that degradation during the 2.5 h period should be greatly diminished. Constitutive CME is orchestrated by several proteins including the Adaptor protein 2 complex (AP2), which is the major adaptor for constitutive CME (Mettlen et al., 2018). AP2 is a heterotetramer consisting of α-adaptin, β2-adaptin, μ2-adaptin, and σ2-adaptin subunits. AP2 initiates pit formation, recruits cargo into the clathrin coated pit and organizes clathrin assembly on the developing vesicle. AP2 continues to interact with recruited cargo and clathrin as the vesicle matures and internalizes, i.e., until after CME is completed; and, AP2 releases cargo and clathrin when the internalized vesicle uncoats before fusion with the endosome. Kv4.2 has two conserved C-terminal motifs that can be directly bound by AP2 subunits (see section “Discussion”). One possibility is that SUMOylation reduces channel internalization by preventing interactions between Kv4.2 and AP2, thereby preventing Kv4.2g from being recruited into clathrin coated pits. To test this idea, we examined the interaction between Kv4.2 and the α-adaptin subunit of AP2. Kv4.2g + HA-KChIP2a + HA-DPP10c were co-expressed with and without co-transfection of SUMO2 + Ubc9. Standard IP experiments with anti-GFP were performed on HEK cells as previously described (Welch et al., 2019). IP products were resolved with PAGE and western blots were probed with anti-adaptin; the blot was then stripped and re-probed for Kv4.2g (Figure 8A). Preliminary control experiments showed that anti-adaptin produced no signal on western blots containing anti-GFP IPs from parental HEK cell lysates. Thus, anti-GFP does not non-specifically IP endogenous adaptin independent of Kv4.2. The OD for the anti-adaptin signal was divided by the OD for the anti-GFP signal to determine the amount of AP2 that associated with Kv4.2g. Plotting the data for the two treatment groups showed that SUMOylation did not significantly alter the amount of AP2 associated with Kv4.2g (Figure 8B). This suggests that the effect of K579 SUMOylation on Kv4.2g internalization is downstream of cargo recruitment into clathrin-coated pits.

### The Unique Influence of KChIP and DPLP on Kv4.2 SUMOylation

**FIGURE 9:**
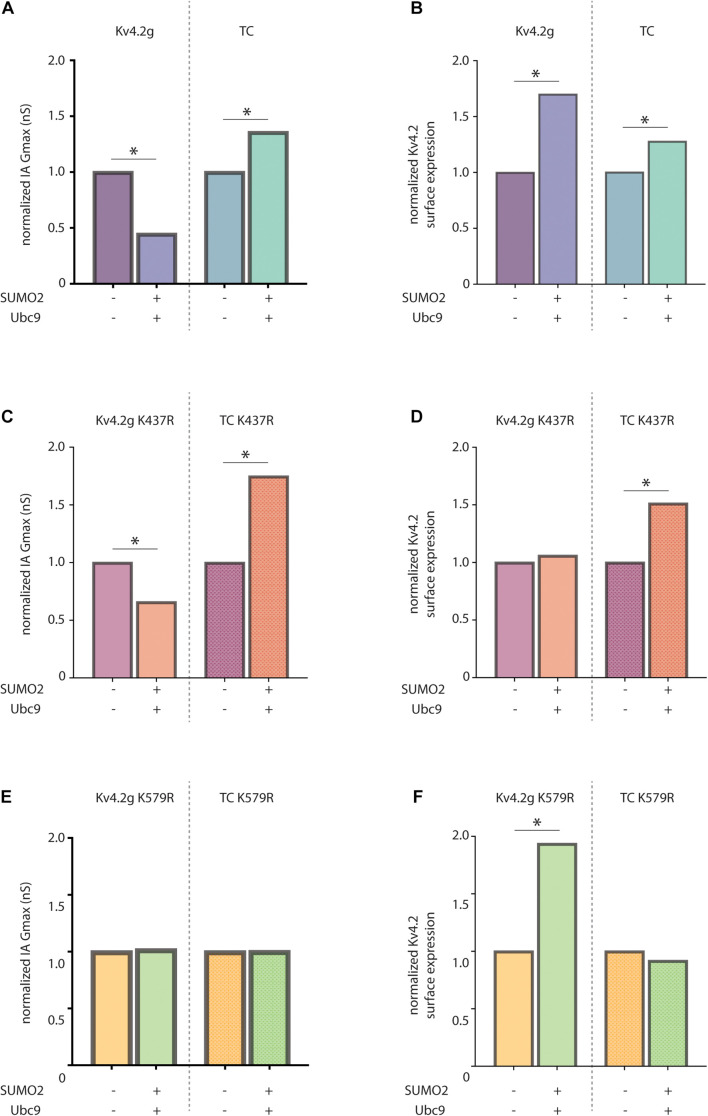
KChIP2a and DPP10c uniquely influence the effects of Kv4.2 SUMOylation. Bar graphs show SUMOylation-induced fold-changes in mean IA Gmax **(A)** or mean surface expression **(B)** in HEK cells expressing only Kv4.2g or all 3 components of the wild-type TC. For each transfection group, e.g., either Kv4.2g or TC, means for the two treatment groups, e.g., ±SUMO2 + Ubc9, were divided by the mean for the treatment lacking SUMO + Ubc9. Bar graphs show mean normalized IA Gmax **(C,E)** or mean normalized surface expression **(D,F)** in HEK cells expressing the indicated Kv4.2g mutant or Kv4.2g mutant TC with (+) and without (–) co-expression of SUMO2 + Ubc9. Asterisk, significantly different. Data for Kv4.2g lacking KChIP2a and DPP10c were adapted from Welch (2019).

The effect of Kv4.2 SUMOylation varies depending upon whether it is heterologously expressed alone or co-expressed with KChIP2a and DPP10c in HEK cells. Enhanced SUMOylation elicited a decrease vs. increase in IA Gmax in HEK cells that expressed Kv4.2g alone vs. Kv4.2g + HA-KChIP2a + HA-DPP10c, respectively (Figure 9A). Enhancing SUMOylation also increased Kv4.2g surface expression to different extents when Kv4.2g was expressed alone or co-expressed with auxiliary subunits (Figure 9B). We ascertained the specific effect of SUMOylation at K437 and K579 under each condition.

SUMOylation of K437 had no effect on IA Gmax under either condition, i.e., SUMOylation still produced significant changes in IA Gmax when K437 was mutated to R (compare Figure 9C vs. Figure 9A). On the other hand, SUMOylation of K437 enhanced Kv4.2g surface expression when it was expressed alone, but not when it was co-expressed with KChIP2a + DPP10c, i.e., the increase in surface expression was blocked by mutating K437 only when Kv4.2g was expressed alone (compare Figure 9D vs. Figure 9B). Together, these data indicate that SUMOylation of K437 increased the insertion of electrically silent channels/subunits only when the pore-forming α-subunit was expressed alone. It is not clear if K437 can be SUMOylated when Kv4.2g is incorporated into the TC, because mutating K437 did not prevent the SUMOylation induced increase in surface expression (compare Figure 9D, right vs. Figure 9B, right) or IA Gmax (compare Figure 9C, right vs. Figure 9A, right) when the α-subunit was co-expressed with KChIP2a and DPP10c.

**FIGURE 10:**
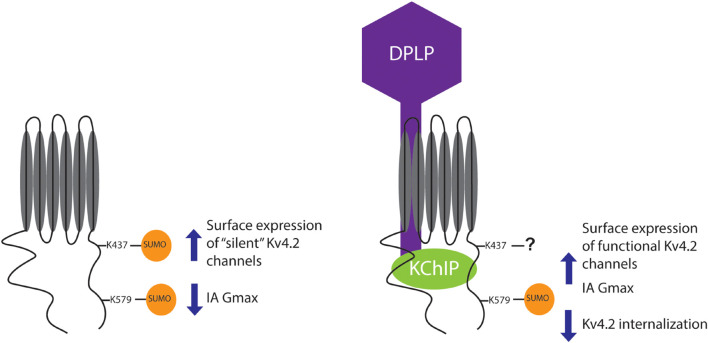
Summary. The cartoon summarizes the effects of Kv4.2 SUMOylation when Kv4.2 is expressed alone versus co-expressed with KChIP2a and DPP10c. Note that the question mark indicates that when Kv4.2 is comprised by the TC, K437 may or may not be SUMOylated, and preventing K437 SUMOylation does not alter the effect of enhanced SUMOylation on channel surface expression and IA Gmax.

SUMOylation of K579 decreased vs. increased IA Gmax when the α-subunit was expressed alone vs. with the other components of the TC, i.e., when K579 was mutated to R, SUMOylation no longer produced a significant change in IA Gmax under either condition (compare Figure 9E vs. Figure 9A). SUMOylation of K579 had no effect on surface expression when the α-subunit was expressed alone, but increased surface expression when Kv4.2g was co-expressed with auxiliary subunits, i.e., when K579 was mutated to R, SUMOylation still produced the significant increase in surface expression when the α-subunit was expressed alone, but not when expressed with TC components (compare Figure 9F vs. Figure 9B). Together, these data showed that in the absence of auxiliary subunits, K579 SUMOylation decreased IA Gmax without altering surface expression. In contrast, in the presence of KChIP2a and DPP10c, K579 SUMOylation increased IA Gmax by reducing internalization (Figures 6, 7). The diagram summarizing the results shows that KChIP2a and DPP10c influence the pattern and/or effect of Kv4.2 SUMOylation (Figure 10).

## Discussion

The biophysical properties of neuronal IA are reproduced in a heterologous system by a TC containing Kv4, KChIP, and DPLP subunits (Jerng and Pfaffinger, 2014). It is not clear if all functional channels exist in a TC in native neurons, and how the TC dynamically interacts with a larger assemblage of variable accessory subunits and regulator proteins as the channel is assembled, trafficked, and modulated in the plasma membrane. Post-translational SUMOylation is likely to play a fundamental role in mediating dynamic interactions with the Kv4.2 TC. This is the first report to examine if/how post-translational SUMOylation modulates the Kv4.2 TC. The current study demonstrated that SUMOylating Kv4.2 K579 reduced internalization and thereby enhanced channel surface expression and IA Gmax. In addition, the effect of Kv4.2 SUMOylation varied with the presence or absence of KChIP2a and DPP10c.

### K579 SUMOylation May Regulate Kv4.2 Surface Expression in Physiologically Relevant Models

Increasing SUMOylation of Kv4.2g at K579 in HEK cells expressing Kv4.2g + HA-KChIP2a + HA-DPP10c produced a significant ∼35–70% increase in IA Gmax. SUMOylation did not orchestrate an increase in IA Gmax by enhancing Kv4.2g protein levels but by producing a significant ∼30–50% increase in channel surface expression. Modulation of K579 SUMOylation status could be a basis for many of the mechanisms regulating Kv4.2 surface expression in a variety of physiologically relevant models. Kv4.2 surface expression can either be increased or decreased following a seizure depending upon the system. In the hippocampus, Kv4.2 surface expression is decreased following kainate-induced status epilepticus (SE) (Lugo et al., 2008; Joshi et al., 2018), while Kv4.2 surface expression is increased following lithium-pilocarpine-induced SE (Joshi et al., 2018). These effects could be mediated by decreasing vs. increasing SUMOylation at K579, respectively. Enhanced excitatory input also regulates Kv4.2 surface expression. Glutamate application to cultured rat cortical or hippocampal neurons results in an increase or decrease in Kv4.2 surface expression, respectively (Lei et al., 2008; Shen et al., 2008). LTP induction or stimulation with AMPA induces Kv4.2 internalization in hippocampal neurons (Kim et al., 2007; Hammond et al., 2008). In some of these examples, post-translational phosphorylation is involved in regulating Kv4.2 surface expression (Hammond et al., 2008; Lugo et al., 2008). This is noteworthy because the phosphorylation state of a protein determines its ability to be SUMOylated (Flotho and Melchior, 2013). For example, activation of PKA prevented the SUMOylation necessary for activity-dependent regulation of IA in an invertebrate neuron (Parker et al., 2019). Preventing K579 SUMOylation should enhance Kv4.2 internalization, and in hippocampal neurons, PKA-dependent phosphorylation of S552 by itself can produce Kv4.2 internalization (Hammond et al., 2008). These data suggest that the S552 phosphorylation state may regulate the K579 SUMOylation state, and thereby, TC surface expression.

### K579 SUMOylation May Regulate Kv4.2 Ubiquitination, and Thereby, Internalization

The surface expression of several membrane proteins is regulated by SUMOylation. SUMOylation of HCN2 (Parker et al., 2017), the CRMP2 subunit of the NaV1.7 channel (Dustrude et al., 2016), mGluR7 (Choi et al., 2016), the dopamine transporter (Cartier et al., 2019), and smoothened (Ma et al., 2016) increased their surface expression, whereas GluR6 SUMOylation decreased its surface expression (Martin et al., 2007). In the 3 cases where the mechanism was examined, post-translational SUMOylation of the protein increased surface expression by reducing ubiquitination of the protein (Dustrude et al., 2016; Ma et al., 2016; Cartier et al., 2019), either by enhancing the protein’s interaction with a de-ubiquitinase (Ma et al., 2016) or by diminishing its interaction with a ubiquitin ligase (Dustrude et al., 2016).

Ubiquitin serves as a trafficking signal recognized by many components of the endocytic and lysosome-targeting machinery (Mettlen et al., 2018; Ferron et al., 2021). In some cases, multiple ubiquitins on a membrane protein may serve as an endocytic signal, and several endocytic adaptors possess ubiquitin binding domains and recruit ubiquitinated cargo into clathrin-coated pits (Piper et al., 2014). Thus, decreasing the ubiquitination of a membrane protein can lead to a decrease in its endocytosis. A ubiquitin signal is also used to regulate membrane protein recycling. After endocytosis and vesicle fusion with the endosome, a membrane protein can either be recycled back to the plasma membrane or it can be recognized by the endosomal ESCRT protein complex and incorporated into vacuoles that remain in the organelle as it matures into the lysosome (Foot et al., 2017; Mettlen et al., 2018). Proteins in the ESCRT system have ubiquitin binding domains that recognize ubiquitin-tagged cargo. Thus, decreasing a membrane protein’s ubiquitination can also lead to an increase in its recycling. To summarize, membrane protein de-ubiquitination generally inhibits internalization by reducing endocytosis and/or increasing recycling.

This study indicated that decorating K579 with SUMO reduced Kv4.2 internalization. The BDM-PUB online prediction tool indicates that Kv4.2 K579 is not a potential site for ubiquitination; and therefore, it is unlikely that K579 is ubiquitinated to produce an endocytic/lysosome sorting signal that promotes internalization and that may be blocked by competitive SUMOylation (Flotho and Melchior, 2013). However, we found that there are seven predicted C-terminal ubiquitination sites on Kv4.2; and, when exogenously expressed in HEK cells, Kv4.2 interacts with several endogenous ubiquitin ligases (ITCH, CAND1, UBE3C, UB20, UBE4B, and UBE2M) as well as de-ubiquitinases (USP9X, USP7, USP24, USP48, USP10, USP10, USP5, and USP15) (Hu et al., 2020). Thus, SUMOylation at K579 could reduce Kv4.2 ubiquitination at a different lysine(s) by enhancing an interaction between Kv4.2 and a de-ubiquitinase and/or by blocking an interaction with a ubiquitin ligase as has been demonstrated in previous studies (Dustrude et al., 2016; Ma et al., 2016; Cartier et al., 2019). Many of the aforementioned ubiquitin ligases and de-ubiquitinases regulate membrane protein internalization including, ITCH (Angers et al., 2004; Tong et al., 2014), UBE4B (Gireud-Goss et al., 2020), USP9X (Kharitidi et al., 2015), USP7 (Han and Yun, 2020), USP10 (Bomberger et al., 2009, 2010), and USP48 (Armando et al., 2014). In addition, post-translational SUMOylation of the de-ubiquitinase, USP5, modulates its association with Cav3.2 α-subunits to regulate channel ubiquitination and the amplitude of the T-type Ca^2+^ current (Garcia-Caballero et al., 2014, 2019).

If enhanced Kv4.2g SUMOylation acts to reduce Kv4.2g ubiquitination at the plasma membrane and/or endosome, as occurred in all previous studies that examined the mechanism (Dustrude et al., 2016; Ma et al., 2016; Cartier et al., 2019), then it will most likely reduce internalization by increasing channel recycling rather than by reducing channel endocytosis. Kv4.2 does not appear to require ubiquitin-binding adaptor proteins for endocytosis because, Kv4.2 can directly associate with AP2 through conserved motifs. The interface between the α/σ subunits of AP2 binds cargo with the [D/E](XXX)_3–5_L[LI] consensus motif (Azarnia Tehran et al., 2019; Beacham et al., 2019), and this motif is present in the C-terminus of Kv4.2 channels (amino acids 475–482). The di-leucine residues in this motif are necessary for Kv4.2 dendritic localization and for activity dependent Kv4.2 internalization (Rivera et al., 2003; Hammond et al., 2008). Kv4.2 also possesses the YXXφmotif that directly binds the μ subunit of AP2 (Mettlen et al., 2018). In addition, our data showed that SUMOylation did not change the amount of binding between Kv4.2 and AP2; and therefore, the effect of SUMOylation on internalization must be downstream of cargo recruitment into the vesicle. Most likely, reduced Kv4.2g ubiquitination would diminish the number of channels recognized and packaged into vacuoles by the ESCRT system; thereby reducing subsequent lysosomal degradation and increasing the number of channels recycled back to the plasma membrane.

### The Unique Influence of KChIP and DPLP on Kv4.2 Post-translational Modifications

KChIP and DPLP proteins can influence Kv4 post-translational modification and its effects. In this sense, the modifications can be said to be context-dependent. Context-dependence is physiologically relevant as α-subunits can associate with a variety of KChIP and DPLP isoforms (Jerng and Pfaffinger, 2014), and recent work suggests that distinct subpopulations of Kv4 channels may exist, wherein Kv4.2 may be dissociated from other components of the TC (Hu et al., 2020). In one example of context dependence, the phosphorylation state of Kv4 is altered by association with KChIP and/or DPLP such that Kv4 is hypophosphorylated when it is expressed alone and hyperphosphorylated when in the TC (Shibata et al., 2003; Seikel and Trimmer, 2009). The effect of post-translational phosphorylation may also be context-dependent. PKA-dependent modulation of Kv4.2 could only be obtained when Kv4.2 was associated with KChIP in a heterologous oocyte expression system (Schrader et al., 2002) and in cultured hippocampal neurons (Prechtel et al., 2018). The effect of Kv4.2 phosphorylation can also vary with the KChIP isoform (Lin et al., 2010).

In this study we showed that KChIP2a and DPP10c can alter the pattern and/or the effect of Kv4.2 SUMOylation. When Kv4.2 is expressed alone, SUMOylation of K579 blocks an unknown interaction to reduce IA Gmax without altering surface expression (Welch et al., 2019); but, when co-expressed with KChIP2a and DPP10c, SUMOylating K579 increased surface expression and IA Gmax. K579 SUMOylation may block a distinct protein–protein interaction when Kv4.2 is in the TC, such as an interaction between Kv4.2 and a ubiquitin ligase. This would imply that different ancillary proteins associate with Kv4.2 when it is in the ternary complex vs. when the channel comprises only α-subunits. KChIP2a and DPP10c co-expression also altered the pattern and/or effect of SUMOylation at K437. When Kv4.2 was expressed alone, enhancing SUMOylation at K437 increased the surface expression of electrically silent channels (Welch et al., 2019); but when Kv4.2 was comprised by the ternary complex, mutating K437 did not alter the effect of enhanced SUMOylation. K437 could be either unSUMOylatable when in the TC or maximally SUMOylated and not enhanceable. Alternatively, enhanced SUMOylation at K437 may no longer regulate surface expression.

KChIP and DPLP may regulate Kv4 SUMOylation status by influencing the Kv4 phosphorylation pattern, perhaps by promoting trafficking to the plasma membrane. In addition, α-subunit folding may be altered by interaction with KChIP and DPLP, which could make sites of post-translational modification more or less available. Similarly, the Kv4 interactome could influence access to SUMOylation sites through, for example, steric hindrance. These same types of phenomena could circumscribe distinct sets of ancillary partners for Kv4 and thereby alter the effects of the post-translational modifications.

## Conclusion

In sum, the effect of Kv4.2 SUMOylation varies depending on the available interactome. When expressed with TC components in HEK cells, Kv4.2g SUMOylation at K579 reduces channel internalization and increases Kv4.2g surface expression and IA Gmax. Future experiments will examine whether SUMOylation increases Kv4.2g recycling by regulating its ubiquitination status by either blocking the channel’s association with a ubiquitin ligase and/or facilitating an interaction with a de-ubiquitinase.

## Data Availability Statement

The original contributions presented in the study are included in the article/Supplementary Material, further inquiries can be directed to the corresponding author.

## Author Contributions

MW and DB were responsible for the conception and design of the work presented. All authors contributed to acquiring and/or analyzing the data. MW drafted the manuscript. All authors were involved in revising the manuscript.

## Conflict of Interest

The authors declare that the research was conducted in the absence of any commercial or financial relationships that could be construed as a potential conflict of interest.

## Publisher’s Note

All claims expressed in this article are solely those of the authors and do not necessarily represent those of their affiliated organizations, or those of the publisher, the editors and the reviewers. Any product that may be evaluated in this article, or claim that may be made by its manufacturer, is not guaranteed or endorsed by the publisher.

## Footnotes

1
www.proteinatlas.org
