# Supplementary material for: SUMOylation of the Kv4.2 Ternary Complex Increases Surface Expression and Current Amplitude by Reducing Internalization in HEK 293 Cells
Source: Front Mol Neurosci. 2021 Nov 2;14:757278. doi: 10.3389/fnmol.2021.757278 (PMC8593141; doi:10.3389/fnmol.2021.757278)
Supplement: Supplementary file 1 [file Image_1.pdf]

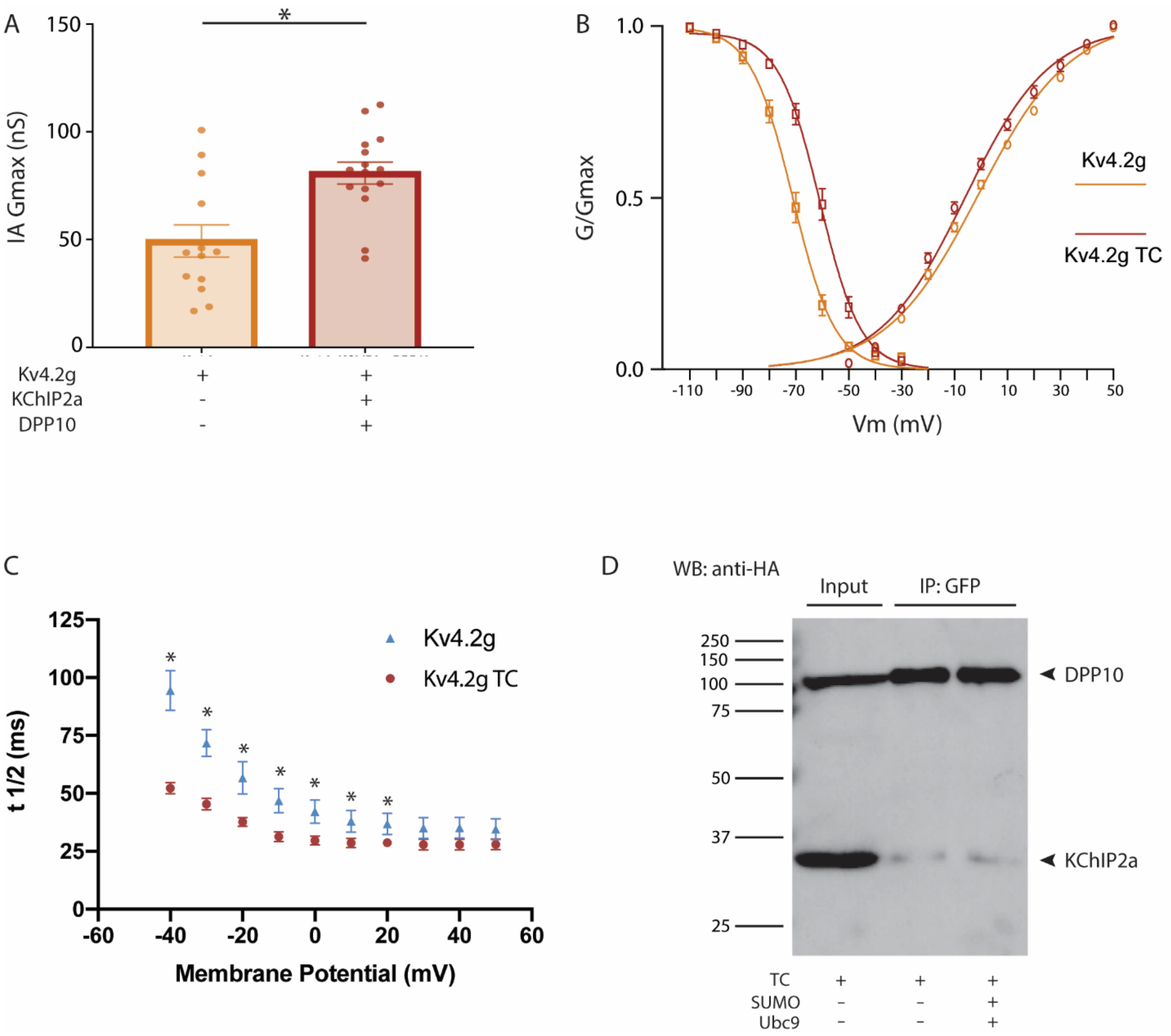

Supplemental Figure 1

(A-C) HEK cells were transiently transfected with Kv4.2g, with or without HA-KChIP2a + HA-DPP10c, followed by whole cell patch clamp. **(A)** IA Gmax significantly increased when Kv4.2 was incorporated into the ternary complex, asterisk, p=0.0015, t-test. mean+SEM: Kv4.2, 49.4+7.4nS; TC, 80.9±5.1nS. **(B)** IA voltage dependence significantly shifted when Kv4.2 was incorporated into the ternary complex. V50 act: p=0.0008, t-test, mean+SEM Kv4.2, 1.0+1.3mV; TC, -5.8+1.2mV. V50 inact: p<0.0001, t-test: mean+SEM Kv4.2, -71.2+1.5mV, n=13; TC, -60.8+1.4mV, n=16. **(C)** Inactivation significantly accelerated when Kv4.2g was incorporated into the ternary complex. Asterisk, p<0.036, t-test, Kv4.2 n=7, TC n=15. **(D)** HA-KChIP2a and HA-DPP10c co-IP with Kv4.2g. HEK cells were transfected with Kv4.2g + HA-KChIP2a + HA-DPP10c. Cells were lysed in IP lysis buffer and Kv4.2g channels were immunoprecipitated with anti-GFP. Lysate (14 µg) and IP product were resolved with PAGE and transferred to a PVDF membrane. The membrane was probed with anti-HA. Arrow at ~100kD is HA-DPP10c and arrow at ~31kD is HA-KChIP2a. Note that KChIP and DPLP do not interact and do not co-IP in the absence of Kv4.2 (Jerng et al., 2005). The intensities of the bands do not reflect stoichiometries. Conditions were not optimized to preserve the interaction between Kv4.2g and HA-KChIP2a or HA-DPP10c in the IP, and western blot transfer times were not optimized. This

blot is only meant to demonsrate interactions between Kv4.2 and the other two members of the ternary complex, n=2.
